# The significant immune escape of pseudotyped SARS-CoV-2 variant Omicron

**DOI:** 10.1080/22221751.2021.2017757

**Published:** 2021-12-21

**Authors:** Li Zhang, Qianqian Li, Ziteng Liang, Tao Li, Shuo Liu, Qianqian Cui, Jianhui Nie, Qian Wu, Xiaowang Qu, Weijin Huang, Youchun Wang

**Affiliations:** a Division of HIV/AIDS and Sex-transmitted Virus Vaccines, Institute for Biological Product Control, National Institutes for Food and Drug Control (NIFDC), Beijing, People’s Republic of China; b Jiangsu Recbio Technology Co., Ltd., Taizhou, People’s Republic of China; c Graduate School of Peking Union Medical College, Beijing, People’s Republic of China; d Translational Medicine Institute, The First People’s Hospital of Chenzhou, University of South China, Chenzhou, People’s Republic of China

**Keywords:** SARS-CoV-2, BA.1, neutralization, convalescence serum, VOC

## Abstract

The emergence of Omicron/BA.1 has brought new challenges to fight against SARS-CoV-2. A large number of mutations in the Spike protein suggest that its susceptibility to immune protection elicited by the existing COVID-19 infection and vaccines may be altered. In this study, we constructed the pseudotyped SARS-CoV-2 variant Omicron. The sensitivity of 28 serum samples from COVID-19 convalescent patients infected with SARS-CoV-2 original strain was tested against pseudotyped Omicron as well as the other variants of concern (VOCs, Alpha, Beta, Gamma, Delta) and variants of interest (VOIs, Lambda, Mu). Our results indicated that the mean neutralization ED50 of these sera against Omicron decreased to 66, which is about 8.4-folds compared to the D614G reference strain (ED50 = 556), whereas the neutralization activity of other VOC and VOI pseudotyped viruses decreased only about 1.2–4.5-folds. The finding from our in vitro assay suggest that Omicron variant may lead to more significant escape from immune protection elicited by previous SARS-CoV-2 infection and perhaps even by existing COVID-19 vaccines.

## Introduction

Since 2019, the COVID-19 pandemic has brought tremendous impact on human life worldwide. The fast development and global distribution of effective vaccines and the breakthrough of small molecule and monoclonal antibody-based drug development have brought light to the prevention and control of this disease. However, on 24 November 2021, a new SARS-CoV-2 variant B.1.1.529/BA.1 was reported, which caused a great disturbance all over the world [1]. Only after two days, on 26th November, the World Health Organization (WHO) Technical Advisory Group on SARS-CoV-2 Virus Evolution designated B.1.1.529 as the fifth variant of concern (VOC), and named it Omicron [2]. Omicron was first detected in a specimen collected in Botswana on 9 November 2021 [1]. The case number increased steeply in Gauteng, South Africa in a short time [1]. As of November 30th, Omicron has been reported in 17 countries and regions, including South Africa, the UK, Australia, Canada, USA, Hong Kong, and the list is growing every day.

Omicron has by far the largest number of mutations among all SARS-CoV-2 variants. In particular, there are 32 mutations located within Spike protein, which is the key viral component that determines the infectivity and antigenicity of the virus [3]. Furthermore, 15 of 32 mutations located right at the receptor binding region (RBD) of Spike protein [3]. These mutations cover almost all the key mutations of the previous VOCs (Alpha, Beta, Gamma, and Delta), including K417N, E484A, and N501Y and other known mutations which are proved to change the sensitivity of the virus to neutralization by protective antibodies [4,5]. It was suggested that the complicated mutations in Spike may lead to escape from immunity induced by prior infection or vaccination, and may cause a large number of breakthrough infection or re-infection with mutated viral strains. In the current study, the pseudotyped SARS-CoV-2 Omicron variant (PV-Omicron) was constructed by using VSV vector and Omicron spike protein containing all of the 32 mutations. The neutralization sensitivity of PV-Omicron against serum samples from a panel of COVID-19 convalescent patients was tested, using PV-D614G (S protein from SARS-CoV-2 D614G strain) as the reference. Other PVs expressing S proteins from current VOCs (Alpha, Beta, Gamma, and Delta) and VOIs (Variants of Interest, Lambda, and Mu) were included as the controls to gauge the relative levels of neutralization for Omicron strain among all key SARS-CoV-2 variants identified until now.

## Results

**Figure 1.**
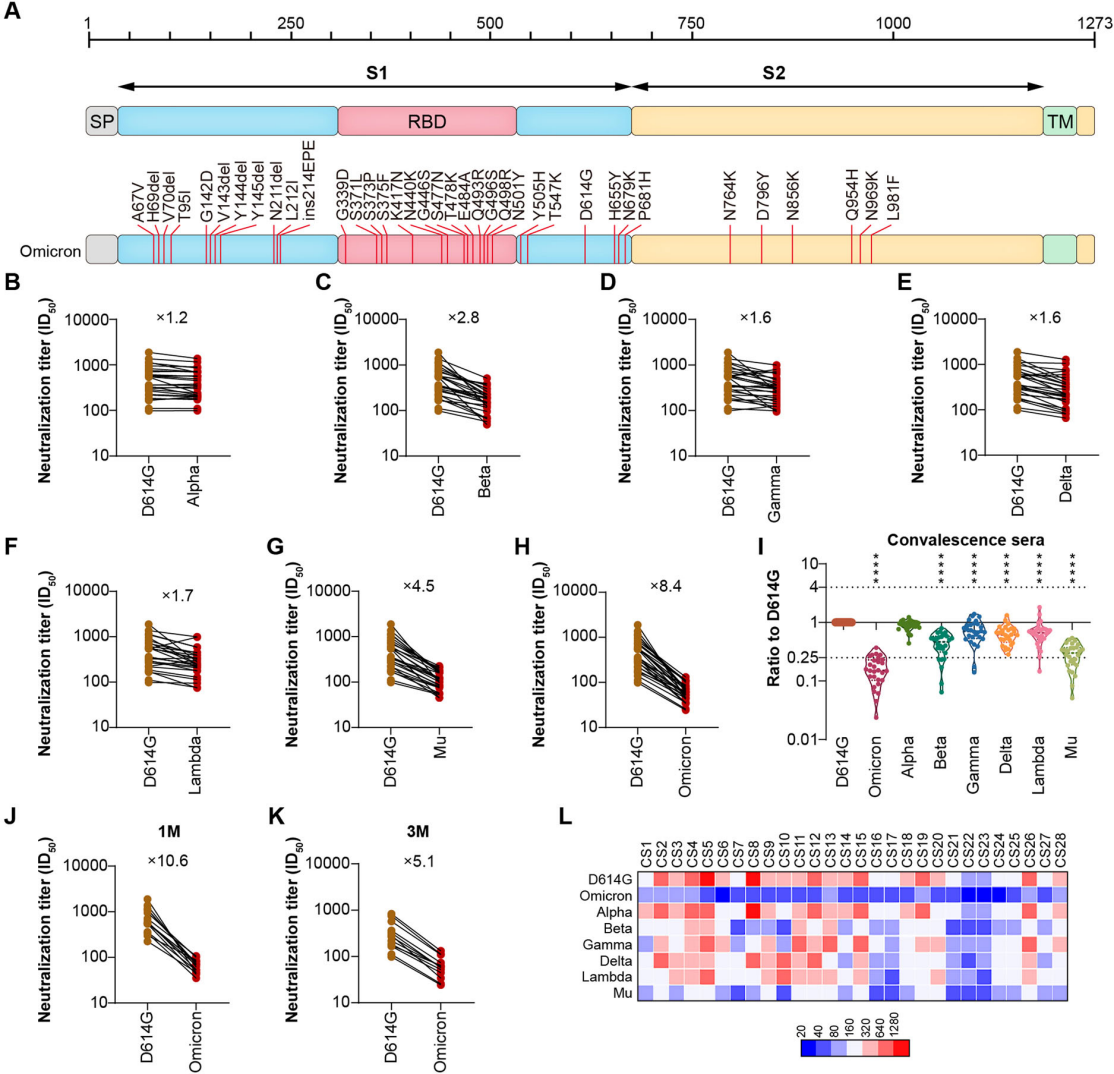
A. Schematic Illustration of Omicron Spike. All of the 32 mutations were located on Omicron Spike, which was used to construct the pseudotyped Omicron virus. B–H. The neutralization analysis of convalescent sera against Omicron, VOCs and VOIs. The neutralization activity of 28 convalescent sera from COVID-19 patients was tested. The neutralization ED50 and ratio compared to the reference strain D614G was also displayed as indicated. Data represented ED50 of three independent experiments. I. The comparison of neutralization activity against different VOCs and VOIs. J–K. The neutralization sensitivity of sera collected from convalescent patients at different time point against Omicron. The neutralization ED50 and ratio compared to the reference strain D614G was also displayed as indicated. Data represented ED50 of three independent experiments. J sera were collected 1-month after recovery; K, sera were collected 3-month after recovery; L. The heatmap of individual neutralization data. Data represented ED50 of three independent experiments. The Red to blue colour represented ED50 high to low as shown in the scale bar. CS1–15 sera were collected 1-month after recovery; CS16–28 sera were collected 3-month after recovery.

There are 32 mutations on the Spike of Omicron, including the following sites: A67V, H69del-V70del, T95I, G142D-V143del-Y144del-Y145del, N211del-L212I, ins214EPE, G339D, S371L, S373P, S375F, K417N, N440K, G446S, S477N, T478K, E484A, Q493R, G496S, Q498R, N501Y, Y505H, T547K, D614G, H655Y, N679K, P681H, N764K, D796Y, N856K, Q954H, N969K, and L981F. The novel pseudotyped virus expressing Spike of Omicron (PV-Omicron) containing all of 32 mutations was constructed (Figure 1A). In addition, pseudotyped viruses expressing S proteins from the other current VOCs (Alpha, Beta, Gamma, and Delta) and VOIs (lambda, mu) constructed as previously were tested and reported [6,7]. To examine how much the neutralization sensitivity of Omicron variant is changed compared to the other SARS-CoV-2 variants, the PV-Omicron was tested against a panel of human sera from the convalescent COVID-19 patients who had infected with the original Strain (Figure 1B–I). The results indicated that the mean neutralization titre (50% effective dilution, ED50) of PV-Omicron was 66, which represented about 8.4-fold reduction of neutralization compared to the reference strain PV-D614G (ED50 = 556) (Figure 1H). Meanwhile, we also examined the neutralization sensitivities of other VOCs and VOIs against the same panel of human serum samples. As for Delta, which is almost the 99% variant right now all over the world, the neutralization activity is only decreased 1.6-fold (Figure 1E). The ED50 of Alpha, Beta, and Gamma is reduced about 1.2, 2.8, and 1.6-fold, respectively, compared to the reference strain PV-D614G (Figure 1B–D), while the reduction of neutralization for Lambda and Mu variants is 1.7- and 4.5-fold, respectively (Figure 1F-G). The heatmap scale showed that there were individual differences among different patient sera against either Omicron or other VOCs groups. As for PV-Omicron, the ED50 has decreased to below 80 for most of the patient sera (Figure 1L). When the serum collected at 1-month or 3-month after recovery was analyzed separately, although the average ED50 of 1-month group (721) is about 2-fold higher than that of 3-month (334) group, they both decreased to the similar lever (ED50 = 68 and 65, Figure 1J-K). Therefore, the decreased neutralization sensitivity against PV-Omicron is more obvious in the 1-month group (10.6-fold) than in the 3-month group (5.1-fold, Figure 1J-K).

## Discussion

Our study is organized to answer the key question that the whole world is anxious to know: will Omicron variant of SARS-CoV-2 escape the existing immunity developed in the global human population in the last 20 months either through natural infection or by mass vaccination? And if the potential escape may occur, to what degree will such escape be observed? Our results showed that the large number of mutations of the Omicron variant did cause significant changes of neutralization sensitivity against a panel of human convalescent sera infected by natural COVID infection, which exceeded all the other previous key variants. The results supported the recent study in South Africa which reported that Omicron was easy to evade immunity from past infection after comparing the epidemiological surveillance data of Beta, Delta with that of Omicron variants [8]. However, although the neutralization sensitivity of convalescent sera decreased quite dramatically, the average ED50 against Omicron is still higher than the baseline, which indicated there is still some protection effect can be observed.

One major caveat of the current study is that we used an in vitro assay system and the pseudotyped viruses but not the real viruses were used in the assay. However, our previous study established a good correlation between our PV-based assay and the assay using real viruses [9]. Additionally, COVID-19 vaccine literature has established that the in vitro neutralization assays are good predictors of vaccine protection efficacy and real-world vaccine effectiveness [10]. Therefore, data from the current PV-based neutralization sensitivity assay may well predict the potential reduction of vaccine protection against the new Omicron variant.

Since the neutralizing antibody titres from both vaccine immunization and SARS-CoV-2 infection decreased gradually over a period of six months, the immune escape of the new variant Omicron may make the situation worse. Although a third-dose enhancement strategy can significantly boost immunity, protection from Omicron may be compromised. In addition, it needs to be re-evaluated whether the therapeutic monoclonal antibodies can still be effective against the Omicron variant. More laboratory and real-world studies are needed to understand whether Omicron can escape from the vaccine-elicited immunity to cause more severe disease and death.

The exact impact on human protection may be influenced by more factors such as the infectivity of Omicron variant relative to other variants to human populations and the viral fitness of Omicron once the humans are infected. More population studies including the level of immune protection and symptoms among people infected with Omicron are needed to fully establish the global impact of Omicron on the control of COVID-19 pandemic.

In summary, this study verified the enhanced immune escape of Omicron variant, which sounds the alarm to the world and has important implications for the public health planning and the development of matching strategies.

## Methods

### Pseudotyped SARS-CoV-2 variants

The Omicron variant gene of SARS-CoV-2 spike protein (GISAID: EPI_ISL_6590782.2) was optimized using mammalian codon and synthesized, then cloned into pcDNA3.1 vector as described previously [11]. The plasmid expressing the S protein of 614G, Alpha, Beta, Gamma, Delta, Lambda, and Mu SARS-CoV-2 variants were previously constructed (Figures S1). The VSV-based pseudotyped SARS-CoV-2 variants was produced by transfecting 293T cells (American Type Culture Collection, CRL-3216) with S protein expression plasmids and simultaneously infected with G*ΔG-VSV (Kerafast, Boston, MA). The titres of pseudotyped viruses were evaluated using Huh 7 (Japanese Collection of Research Bioresources, 0403) cells by 3-fold serial dilutions. Chemiluminescence signals were detected 24 h after the incubation of cells and virus at 37°C with 5% CO_2_. The Britelite plus reporter gene assay system (PerkinElmer, Waltham, MA) and PerkinElmer Ensight luminometer were used for signal collection. The detailed procedure was described in our previous paper [12].

### *In vitro* neutralization assay

Serum samples were 1:30 diluted, followed by a 3-fold serial dilution. The diluted sera were mixed with pseudotyped SARS-CoV-2 variants (1.3 × 10^4^ TCID_50_) in 96-well plates, respectively. The mixture was incubated at 37°C for 1 h, and then mixed with Huh 7 cells. The chemiluminescence signals in terms of relative luminescence unit (RLU) values were determined as described previously. The 50% effective dilution (ED_50_) was calculated using the Reed–Muench method.

### Sera from SARS-CoV-2 convalescent patients

Serum samples from convalescent COVID-19 patients were collected from March to October 2020 by the Institute of Translational Medicine, First People's Hospital of Chenzhou. A total of 28 samples with the relative high neutralization activity in our preliminary study were selected. CS1–15 sera were collected one month after recovery; CS16–28 sera were collected three month after recovery. Consent forms were signed prior to blood collection. Ethical approval was obtained from the Institute of Translational Medicine, University of South China (V1.0, 203301).

## Statistical analysis

Data were plotted using GraphPad Prism 8 (GraphPad, San Diego, CA). One-way ANOVA and Holm–Sidak multiple comparison tests were used for statistical analysis. Significance thresholds: * *p* < 0.05, ** *p* < 0.01, *** *p* < 0.005, and **** *p* < 0.001.

## Supplementary Material

Supplemental Material
