# Supplementary figures and images for: The significant immune escape of pseudotyped SARS-CoV-2 variant Omicron
Source: Emerg Microbes Infect. 2021 Dec 21;11(1):1–5. doi: 10.1080/22221751.2021.2017757 (PMC8725892; doi:10.1080/22221751.2021.2017757)

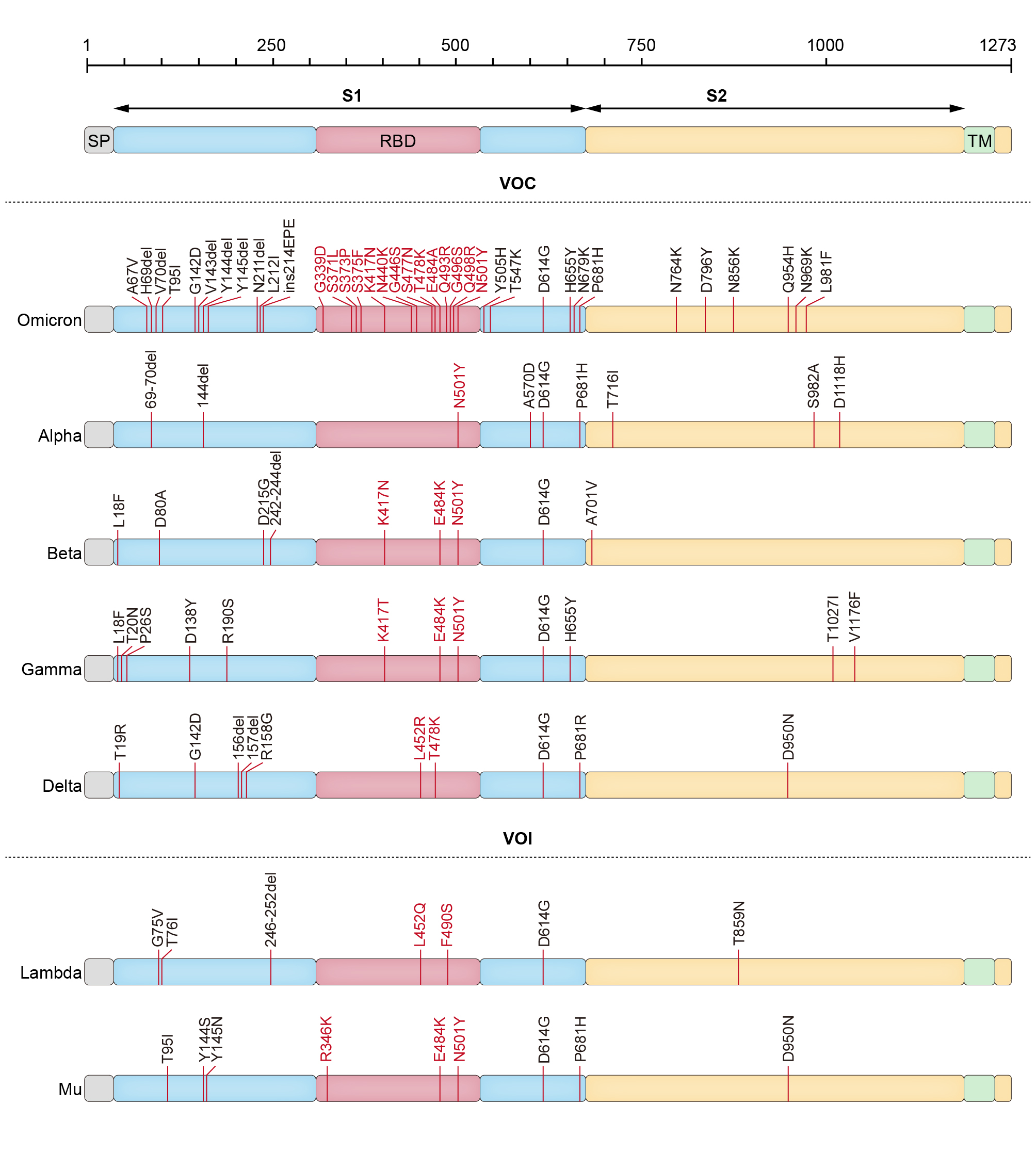

Supplement: Supplemental Material [file TEMI_A_2017757_SM9424.jpg]
